# Risk of Severe Upper Gastrointestinal Complications among Oral Bisphosphonate Users

**DOI:** 10.1371/journal.pone.0073159

**Published:** 2013-12-09

**Authors:** Arianna Ghirardi, Lorenza Scotti, Antonella Zambon, Gianluca Della Vedova, Luca Cavalieri D'oro, Francesco Lapi, Francesco Cipriani, Achille P. Caputi, Alberto Vaccheri, Dario Gregori, Rosaria Gesuita, Annarita Vestri, Tommaso Staniscia, Giampiero Mazzaglia, Giovanni Corrao, on behalf of the BEST Investigators

**Affiliations:** 1 Department of Statistics and Quantitative Methods, Division of Biostatistics, Epidemiology and Public Health, University of Milano-Bicocca, Milan, Italy; 2 Department of Informatics, Systems and Communications, University of Milano-Bicocca, Milan, Italy; 3 Operative Unit of Epidemiology, Local Health Unit of Monza, Monza-Brianza, Italy; 4 Department of Epidemiology, Regional Agency for Healthcare Services of Tuscany, Florence, Italy; 5 Department of Preclinical and Clinical Pharmacology, University of Florence, Florence Italy; 6 Centre for Clinical Epidemiology, Jewish General Hospital, McGill University, Montreal, Quebec, Canada; 7 Department of Medicine and Pharmacology, University of Messina, Messina, Italy; 8 Regional Centre for Drug Evaluation and Information (CREVIF), Department of Pharmacology, University of Bologna, Bologna, Italy; 9 Department of Public Health and Microbiology, University of Turin, Turin, Italy; 10 Center of Epidemiology, Biostatistics, and Medical Information Technology, Polytechnic University of Marche, Ancona, Italy; 11 Department of Experimental Medicine and Pathology, University “La Sapienza”, Rome, Italy; 12 Department of Medicine and Aging, University “G. d'Annunzio”, Chieti-Pescara, Italy; University of Milan, Italy

## Abstract

**Background:**

Oral bisphosphonates (BPs) are the primary agents for the treatment of osteoporosis. Although BPs are generally well tolerated, serious gastrointestinal adverse events have been observed.

**Aim:**

To assess the risk of severe upper gastrointestinal complications (UGIC) among BP users by means of a large study based on a network of Italian healthcare utilization databases.

**Methods:**

A nested case-control study was carried out by including 110,220 patients aged 45 years or older who, from 2003 until 2005, were treated with oral BPs. Cases were the 862 patients who experienced the outcome (hospitalization for UGIC) until 2007. Up to 20 controls were randomly selected for each case. Conditional logistic regression model was used to estimate odds ratio (OR) associated with current use of BPs after adjusting for several covariates. A set of sensitivity analyses was performed in order to account for sources of systematic uncertainty.

**Results:**

The adjusted OR for current use of BPs with respect to past use was 0.94 (95% CI 0.81 to 1.08). There was no evidence that this risk changed either with BP type and regimen, or concurrent use of other drugs or previous hospitalizations.

**Conclusions:**

No evidence was found that current use of BPs increases the risk of severe upper gastrointestinal complications compared to past use.

## Introduction

Osteoporosis is a condition characterized by low bone mineral density and alterations of the microarchitecture of the skeleton that determines fragility of the bone and subsequent increased risk of fracture, even in case of mild traumas [1]. Approximately 75 million subjects in Europe, Japan and USA are affected by osteoporosis [2].

Bisphosphonates (BPs), such as alendronate and risedronate, are considered mainstay therapy for the treatment of osteoporosis. Randomised clinical trials (RCTs) have consistently shown that treatment with these agents improves bone mineral density (BMD) and reduces bone fracture risk [3]–[9]. However, long-term therapy is necessary to increase and maintain BMD and to maintain normal levels of bone resorption [10]. Therefore, therapy must be generally safe, besides being effective, in a long-term fashion. Data from the pivotal RCTs of both alendronate [3]–[5] and risedronate [7]–[9], [11], [12] did not find clinical evidence of adverse effects greater than placebo. However, soon after alendronate release, many cases of oesophageal ulcerations were encountered, so resulting in changes to the alendronate label [13], [14]. From then on nowadays, inconsistent findings on gastrointestinal (GI) safety of BPs have been reported [15]–[20]. Two meta-analyses on this topic came to conflicting conclusions [21], [22], suggesting that evidence are still insufficient to assess the gastrointestinal safety of these agents.

The aim of this nested case-control study was to assess the relationship between current use of BPs and the risk of hospitalization for severe UGIC. Controlling for sources of systematic uncertainty was of particular concern in this study.

## Methods

### Data source

Italian population is covered by the National Health Service (NHS). The healthcare service delivered by NHS to its beneficiaries is associated with an automated system of databases including: (i) an archive of residents who receive NHS assistance (i.e. the whole resident population), reporting demographic and administrative data, as well the dates of starting and stopping to benefit from NHS assistance; (ii) a public and private hospital discharge database; and (iii) a database on outpatient drug prescriptions reimbursable by the NHS.

The primary sources of data were the databases of the 13 Italian territorial units participating at the AIFA-BEST project. This last is a National collaborative study funded by the Italian Agency of Drug (AIFA) which was aimed of assessing BPs safety profile in the Italian clinical practice. Territorial units were four Regions (Abruzzo, Emilia-Romagna, Marche and Toscana) and nine Local Health Authorities (Caserta, Como, Gorizia, Latina, Lodi, Milano, Monza, Sondrio and Varese). A population of about 17 million of beneficiaries of NHS residents in these territorial units was covered by the corresponding databases, accounting for nearly 30% of the whole Italian population.

Hospital discharge diagnoses and drug prescriptions of each patient were assessed through a record linkage procedure based on the unique individual identification code (Regional Health Code) consistently reported in all databases. In order to preserve privacy, we replaced the original identification code with its digest that is the image of the code through a cryptographic hash function – the Secure Hash Algorithm (SHA-256). Such hash function makes infeasible to obtain the original code from the digest, is deterministic (i.e. the same digest is always associated to any given individual) and collision-resistant (the probability that two individuals are associated to the same code is insignificant). The specific hash function used (SHA-256) is the industry standard [23] and has been incorporated into the data extraction-transformation-load software produced by the University of Milano-Bicocca.

All data were drawn out by means of standardized queries which were discussed and agreed upon in conference together with the study protocol. Appendix S1 provides specific diagnostic therapeutic codes used in our study.

### Study cohort

The target population included all beneficiaries of NHS residents in the above mentioned territorial units aged 45 years or older. According to the 2001 Italian Census, this population comprised 6,135,458 individuals. Of these, those who received at least one dispensation of BP reimbursable by the NHS (alendronate and risedronate) from July 1, 2003 until December 31, 2005 were identified, and the date of first dispensation was designed as initial prescription.

Exclusion criteria regarded patients who, within six months before the initial prescription, (i) BPs were already been dispensed (in order of favouring the inclusion of newly treated individuals), (ii) were hospitalized for osteoporotic fractures (in order to focus on primary prevention of osteoporotic fractures), (iii) were hospitalized for gastrointestinal adverse events (in order to focus on incident cases of UGIC during follow-up) or with diagnosis of coagulation disorders, alcohol abuse, chronic liver disease, or cancer (in order to exclude higher-risk patients). In addition, patients who were registered as NHS beneficiaries from less than six months prior the initial prescription, and those who did not reach at least two months of follow-up, were also excluded to ensure a sufficient time-window of wash out and of exposure to the drugs of interest, respectively. The remaining patients constituted the study base population.

Each member of the cohort accumulated person-years of follow-up from the date of study entry until the earliest date among those of outcome onset (hospital admission for UGIC) or censoring (death, emigration or December 31, 2007).

### Selection of cases and controls

A case-control study was nested into the cohort of BPs users. Cases were the cohort members who during follow-up experienced at least a hospitalization with primary or secondary diagnosis of UGIC including: oesophageal/gastrointestinal ulcer, perforation of oesophagus, oesophageal/gastrointestinal haemorrhage (see Appendix S1). The earliest date of hospital admission recording one of these events was considered as the index date.

Up to twenty controls for each case were randomly selected from the cohort members to be matched for territorial unit of recruitment, gender, age at study entry, date of initial prescription and who survived at least as long as the index case. The index date of each matched control was fixed as the same as the index date of his case patient.

### Exposure assessment

During the study period two drug types (alendronate and risedronate) either on once-daily (10 mg/day and 5 mg/day, respectively) or once-weekly (70 mg/week and 35 mg/week, respectively) regimens were available for free reimbursement by Italian NHS.

The length time with drug available for each refilled canister was calculated assuming the standard frequency of intake and the prescribed dosing regimen (i.e. two or four weeks). The date of exposure stopping of each dispensation was accordingly established. For overlapping prescriptions, individuals were assumed to have refilled early and completed the first prescription before starting the second. Exposure to BPs was categorized as “current,” when the supply of the most recent prescription lasted until the index date or ended in the 30-day period prior the index date or “past”, when the latest use was in the 31 days or more prior the index date. Current users were further classified according to type and regimen of the latest dispensed BP.

Because we had no information about drug prescriptions for inpatients, with the aim of avoiding the so called immeasurable time bias, i.e. the differential misclassification due to unmeasured drug exposure during hospitalizations [24], the observation period was temporarily censored at the date of hospital admission for a cause that differed from UGIC, and re-established 10 days after hospital discharge.

### Covariates

For each case and control the occurrence of hospitalizations (for any diagnosis that differed from UGIC), as well as the use of other drugs over the 60-day period prior the index date were investigated. Other drugs included antidepressants, antithrombotic, gastroprotective agents, corticosteroids, statins, calcium channel blockers, other antihypertensive agents and nonsteroidal antiinflammatory agents (NSAIDs) (see Appendix S1).

### Conventional analyses

Chi-square, or its version for the trend, was used when appropriate to test the differences between cases and controls. A conditional logistic regression model was fitted to estimate the odds ratio (OR), as well as its 95% confidence interval (CI), of UGIC in relation to current use of BPs, with respect to past use. Adjustments were made for the above reported covariates.

The combined effects of current BPs exposure together with use of other drugs, occurrence of hospitalizations and type and regimen of the dispensed BP, were estimated by including the corresponding interaction terms in a conditional logistic model.

### Sensitivity analyses

The following five sets of sensitivity analyses were performed.

First, we verified if our estimates were affected by the adopted criteria for defining UGIC. Data were analysed according to alternative diagnostic criteria, i.e. those recently proposed by *Cadarette* & coll. while investigating oral BPs safety [20], as well as those used by a collaborative project aimed to exploit European healthcare databases for drug safety signal detection, the so called EU-ADR project [25].

Second, we verified if our estimates were affected by the adopted criteria for defining current exposure. Data were analyzed according to time-window of 7, 15 or 45 days prior the index date for defining current use, alternative to 30 days as in the main analysis.

Third, we verified if our estimates were affected by protopathic bias, i.e. if the use of BPs among cases could have been attenuated in the current period owing to the onset of early symptoms of UGIC [26]. To control for such a bias, lag-time of 7, 15, 30 or 45 days prior the index date were used before starting the backward clock for measuring current exposure.

Fourth, the robustness of our findings regarding potential bias introduced by the inclusion into the cohort of prevalent BP users was investigated. Let the observed BPs – UGIC association be expressed by the sum of the BPs – UGIC associations among prevalent and incident users weighted by the corresponding prevalence. Hence, if only incident users had been included, the true BPs – UGIC association would be estimated from the following two quantities: (i) the proportion of prevalent BP users possibly included into the investigated cohort (p); (ii) the BPs – UGIC association expected among prevalent users. The proportion of cohort members who had at least one BP prescription within four years before the initial prescription (rather than within six months as in the main analysis) was used to estimate the first quantity. These data were only available for a subcohort of patients recruited by some Local Health Authorities (Como, Lodi, Milano, Monza, Sondrio and Varese). We subsequently allowed the possible values of the BPs – UGIC association among prevalent users to vary from 0.2 to 1 (i.e. with respect to no BP users in the current period, those who currently used BPs may have experienced UGIC hospitalization up to 5-fold less).

Fifth, the robustness of our findings with regard to potential bias introduced by unmeasured confounders was investigated by using the rule-out approach described by *Schneeweiss* [27]. In applying the rule-out method, we allowed the possible unmeasured confounder (i) to be associated with the outcome with risk ratio varying from 1 to 10 (i.e. the exposed patients to this factor may have experienced hospitalization for UGIC up to 10-fold more, or less, than unexposed), (ii) to be associated with the exposure of interest with odds ratio varying from 1 to 10 (i.e. current users of BPs may be exposed to this factor up to 10-fold more, or less, than patients who currently do not use BPs), and (iii) to be present in the study population with a prevalence 10%, 25% or 50%. In its original formulation, rule-out approach aims to detect the extension of confounding required to fully account for the observed exposure-outcome association so moving the observed point estimate to the null. In our application, we generalized the use of the rule-out approach at the situations in which the observed association did not reach statistical significance and the interest was to detect the extension of confounding required to make statistically significant the observed exposure-outcome association. With this aim, we conducted the analysis for the value of the observed lower 95% confidence limit to determine the constellations in which the 95% confidence interval would not cross the expected value under the null hypothesis.

The SAS statistical package was used for the analyses (SAS, Version 9.1; SAS Institute, Cary, North Carolina, USA). For all hypotheses tested two-tailed p-values less than 0.05 were considered to be significant.

### Ethical considerations

According to the rules from the Italian Medicines Agency (available at: http://www.agenziafarmaco.gov.it/sites/default/files/det_20marzo2008.pdf) retrospective studies without direct contact with patients do not need a written consent to process personal data when they are used for research aims. AIFA and the Independent Ethics Committees (IEC) of all the territorial units involved in the investigation (please, see Appendix S2 for the complete IEC list) approved the study protocol.

## Results

### Sample selection

**Figure 1:**
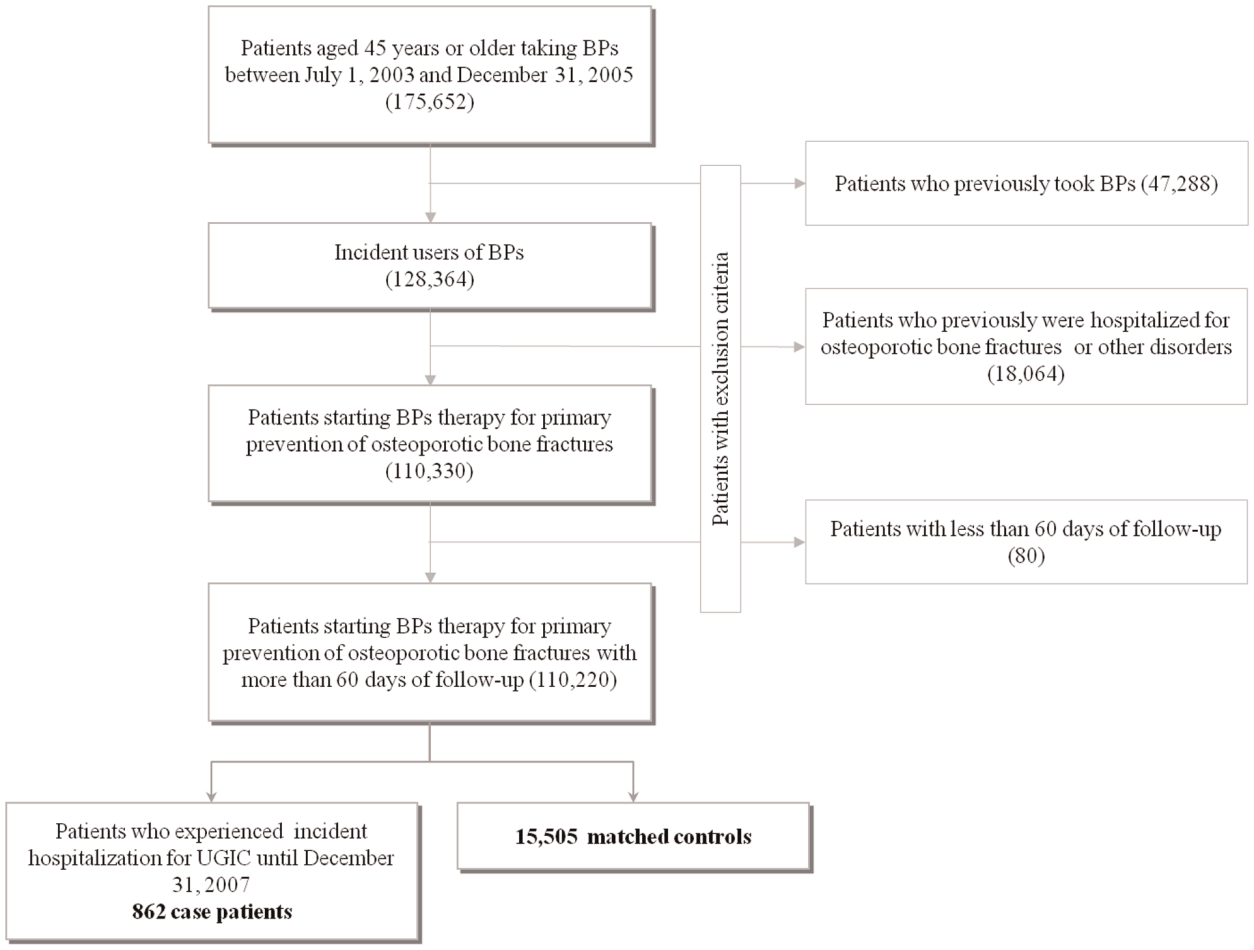
Study flow-diagram. AIFA-BEST project, Italy, 2003–2007. BPs: Bisphosphonates.

The distribution of the exclusion criteria is shown in Figure 1. At entry, the 110,220 patients who were included into the study had mean age of 70.9 years (SD 10.3) and 86.1% of them were women. During follow-up these patients accumulated 335,845 person-years of observation and generated 862 hospital admissions for UGIC, with an incidence rate of 26 cases per 10,000 person-years. The 862 patients who experienced hospitalization for UGIC (case patients) were matched to 15,505 controls.

### Patients

At the date of the initial prescription, mean age of cases and controls was almost 76.7 years (SD: 8.6), and nearly 81% of them were women (matching variables). As shown in Table 1, current use of BPs was more frequent among controls than cases. Conversely, case patients used more frequently the other considered drugs (with the exception of statins and calcium channel blockers) and also experienced more hospitalizations than controls.

**Table 1:** Selected tracts of the 862 cases of upper gastrointestinal complications and 15,505 matched controls.

	Case patients	Controls	p-value*
**Drug use**			
Bisphosphonates†	419 (48.6%)	8,055 (52.0%)	0.0559
Antidepressants‡	133 (15.4%)	1,680 (10.8%)	<0.0001
Antithrombotic‡	245 (28.4%)	3,317 (21.4%)	<0.0001
Gastroprotective agents‡	241 (28.0%)	2,867 (18.5%)	<0.0001
Corticosteroids‡	119 (13.8%)	1,046 (6.8%)	<0.0001
Statins‡	70 (8.1%)	1,549 (10.0%)	0.0735
Calcium channel blockers‡	136 (15.8%)	2,115 (13.6%)	0.0763
Other antihypertensive drugs‡	480 (55.7%)	6,990 (45.1%)	<0.0001
Nonsteroidal antiinflammatory drugs‡	250 (29.0%)	2,987 (19.3%)	<0.0001
**Number of hospitalizations** ‡			
0	735 (85.3%)	14,781 (95.3%)	<0.0001
1	103 (11.9%)	640 (4.1%)	
≥2	24 (2.8%)	84 (0.6%)	

† Measured over the 30-day period prior the index date.

‡ Measured over the 60-day period prior the index date. Hospital admissions considered in this count does not include hospitalization for UGIC.

* According to chi-square test or chi-square test for trend (number of previous hospitalizations).

AIFA-BEST project, Italy, 2003–2007.

### Current use of bisphosphonates and the risk of upper gastrointestinal complications

**Figure 2:**
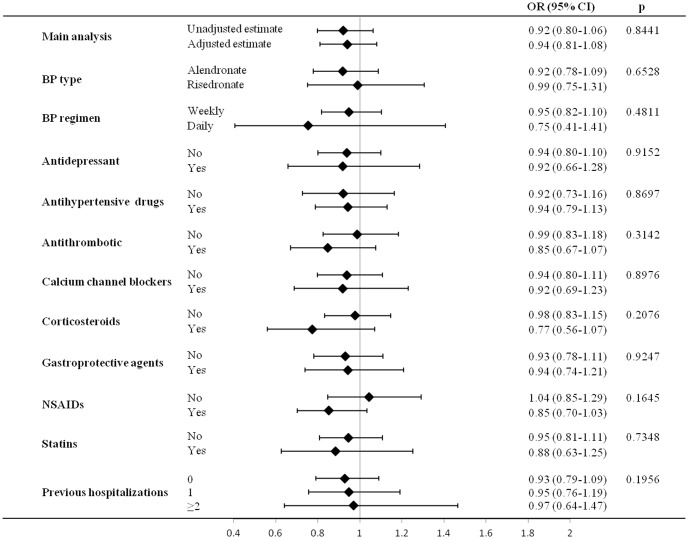
Adjusted odds ratios (and 95% confidence intervals) of upper gastrointestinal complications associated with current use of bisphosphonates within various patient subgroups. AIFA-BEST project, Italy, 2003–2007. Odds ratios estimated with conditional logistic regression model. Estimates concerning main analysis were unadjusted and adjusted for use of other drugs and for the number hospitalizations in the 60-day period prior the index date. Estimates concerning subgroup analysis were obtained by including the interaction terms combining the effect of current use of BPs together with BPs type and regimen dispensed during the current period, concurrent use of other drugs and number hospitalizations in the 60-day period prior the index date. P-values concern comparison of BPs effect across patient subgroups or along increasing number of hospitalizations. BPs: Bisphosphonates.

There was no evidence that current BP users had increased UGIC risk with respect to past users either from unadjusted and adjusted estimates (being the corresponding OR, and 95% CI, respectively, 0.92, 0.80 to 1.06 and 0.94, 0.81 to 1.08, Figure 2). Figure 2 also shows that there was no evidence that the ORs were heterogeneous across patients stratified according with either types or regimens of dispensed BPs, concurrent use of other drugs and number of previous hospitalizations.

### Sensitivity analyses

**Figure 3:**
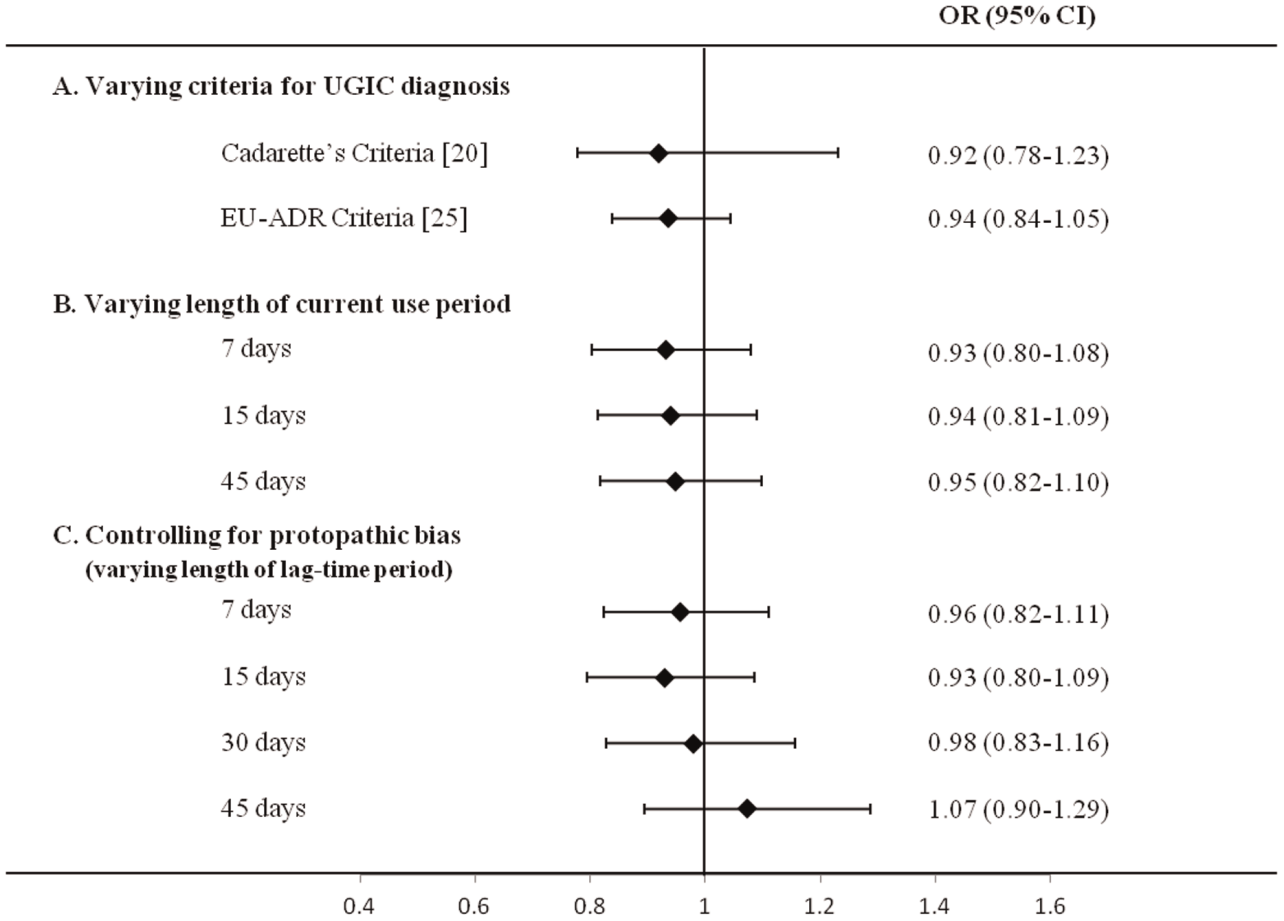
Influences of diagnostic criteria for upper gastrointestinal complications (panel A), length of time-window for current use of bisphosphonates (panel B), and of controlling for protopathic bias (panel C) on the observed odds ratio for upper gastrointestinal complications associated with current use of bisphosphonates. AIFA-BEST project, Italy, 2003–2007. Estimates are adjusted for use of other drugs and number of hospitalizations in the 60-day period prior the index date. Details for diagnostic criteria are reported in Appendix S1. For an explanation of methods for controlling protopathic bias see the “Sensitivity analysis”, subsection of the “Methods” section. BPs: Bisphosphonates.

Figure 3 shows that OR substantially did not change by varying criteria for diagnosis of UGIC (box A) and the length of the time-window defining current BPs use (box B), neither by introducing lag-time periods to take into account possible protopathic bias (box C).

**Figure 4:**
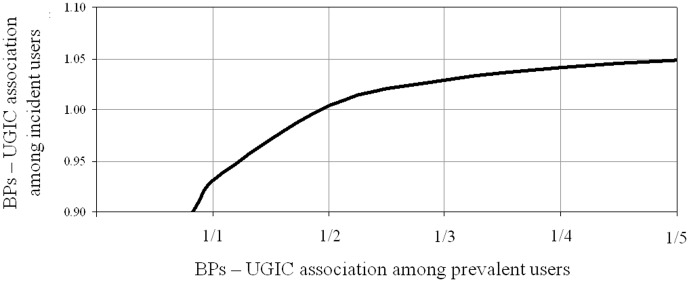
Modelled influence of the inclusion of prevalent BPs users on the true association between current BPs and UGIC risk. AIFA-BEST project, Italy, 2003–2007. The graph indicates the trend of the true effect of BPs current use on the UGIC risk (e.g. the odds ratio which we would have observed if only incident users were included) according to different values of the BPs – UGIC association among prevalent users. For an explanation see the “Sensitivity analysis”, subsection of the “Methods” section. BPs: Bisphosphonates. UGIC: Upper gastrointestinal complication.

Figure 4 shows the robustness of our findings with regard to potential bias by prevalent users. Let us assume a 12.8% proportion of prevalent BPs users, i.e. the prevalence estimated from the subcohort for which these data were available. Furthermore, let us suppose that prevalent users currently exposed to BPs have a two-fold decreased UGIC risk than those unexposed. Then, the true BPs – UGIC association was about 1.0, i.e. the expected value under the null. However, even supposing a much higher apparent (biased) protective BPs effect among prevalent users (e.g. a five-fold reduced risk) a 5% increased risk associated with current BPs use is expected.

**Figure 5:**
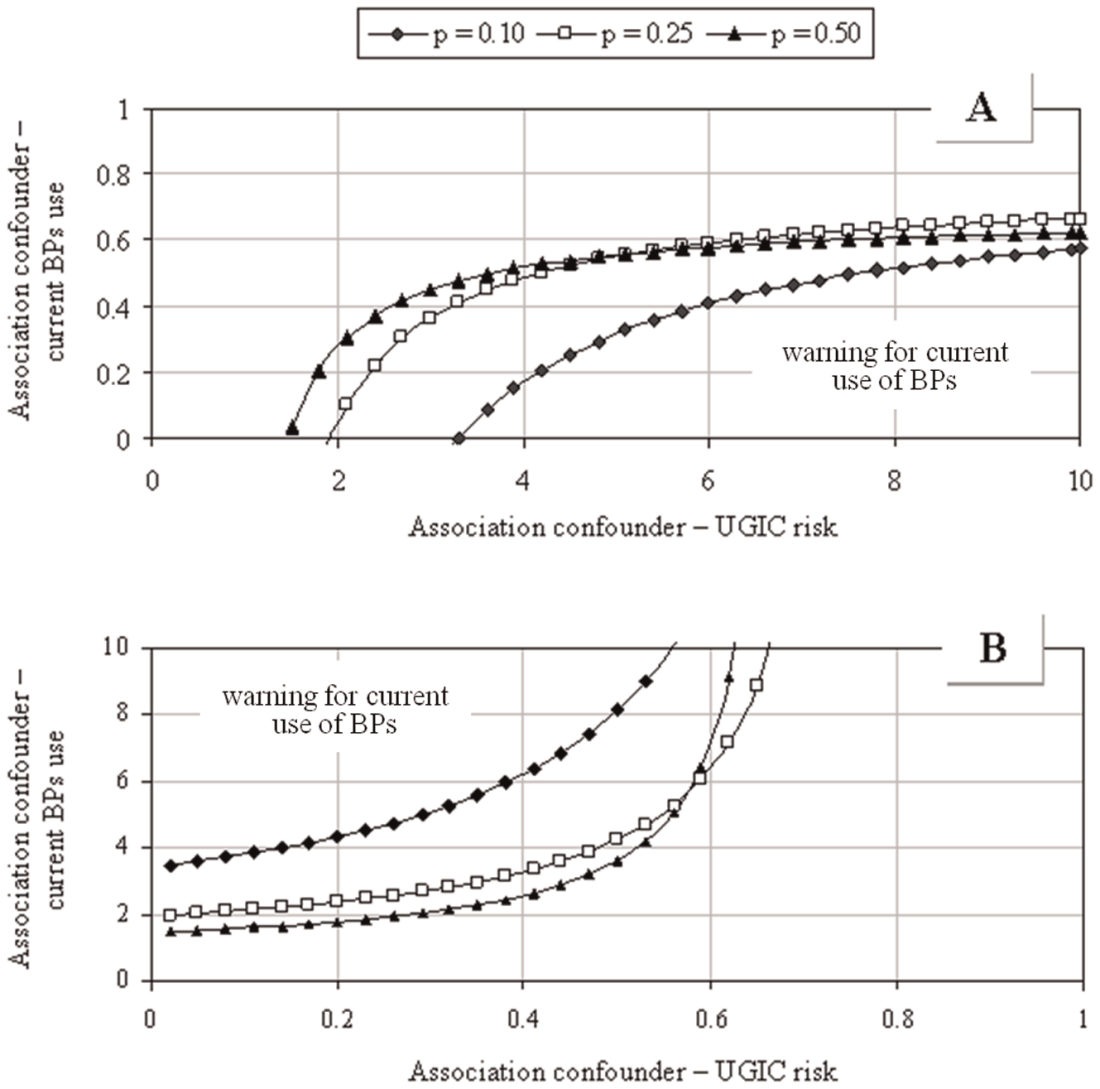
Modelled influence of a hypothetical confounder unaccounted for in the adjustments already performed in the main analysis according with the direction of its effect on the outcome (i.e. positive and negative associations as reported in boxes A and B, respectively), and with its prevalence in the study population (p). AIFA-BEST project, Italy, 2003–2007. The graphs indicate what combinations of confounder – UGIC and confounder – current BPs exposure would be required to make statistically significant the observed association between current use of BPs and hospitalization for UGIC. For an explanation see the “Sensitivity analysis”, subsection of the “Methods” section. BPs: Bisphosphonates. UGIC: Upper gastrointestinal complication.

The results of the residual confounding analysis obtained by means of the rule-out approach are presented in Figure 5. An unmeasured confounder is expected to be positively and negatively associated with UGIC risk in box A and B respectively. Let us assume a 10% prevalence of exposure to a hypothetical unmeasured factor (U). Furthermore, let us suppose that patients exposed to U have a 4-fold increased UGIC risk than those unexposed. In these conditions (Box A), if BPs significantly increased the UGIC risk, then patients exposed to U would reduce their exposure of 5.8-fold or more during the current intake of BPs. A reduction of exposure to U of 2-fold or more would be required: (i) for prevalence of 25% or 50%; (ii) for stronger 8-fold confounder-outcome association. Now, let us assume that patients exposed to U have a 4-fold reduced UGIC risk than those unexposed (Box B). If BPs significantly increased the UGIC risk, then patients exposed to U would increase their exposure of 4.7-fold during the current use of BPs.

## Discussion

In this large nested case-control study we found no evidence of an increased severe UGIC risk associated with current use of BPs compared to past use. Our findings further suggest that there is no important difference in gastrointestinal safety between risedronate and alendronate, neither between daily and weekly regimens. We also found that concurrent therapy with drugs known or suspected to increase the UGIC risk (e.g. antidepressants, antithrombotic, corticosteroids, antihypertensive agents and NSAIDs), as well as previous hospitalizations, did not modify the risk associated with current use of BPs.

### Comparison with literature

Our results are consistent with those of the Fracture Intervention Trial (FIT) which reported a similar proportion of patients who experienced upper GI events in the alendronate and placebo arms, irrespectively of planned drug dose (5 mg or 10 mg) or patients' demographic and clinical characteristics (older age, previous upper GI disease, NSAIDs use) [28]. In addition, our results complied with trials on risedronate. A pooled analysis from 9 RCTs confirmed that the rate of upper GI tract adverse events was similar across risedronate and placebo groups, and that concomitant use of aspirin, NSAIDs, H_2_-receptor antagonists and/or proton pump inhibitors did not lead to significant between-group differences in the incidences of these events [29]. Other RCTs showed inconsistent findings. Three trials found significant higher risk of oesophageal ulcerations among etidronate users [15], and of perforations, ulcerations, or bleeding episodes among etidronate users [15]–[31]. Pooled analyses found no significant effects for other bisphosphonates [32], [33]. Consistently with our findings, observational studies (OSs) did not found evidence of increased UGIC risk among BP users [15], [16], [19]. However, conversely to our results, BPs – NSAIDs co-therapy has been found to increase the UGIC risk [18], [19]. It should be noticed, that our findings were obtained by contrasting current and past use of BPs, rather than BPs use vs. placebo (or vs. no use) as in RCTs (or in OSs). However, assuming an acute effect of BPs on the considered outcome, past users and no users (placebo) would be both suitable comparators for current users.

Validity of our main findings seems to have support by the observed association between use of other drugs and the considered outcome. For example, consistently with literature, we found that, with respect to controls, case patients had higher prevalence in the use of NSAIDs, antihypertensive, antithrombotic and corticosteroid drugs, other than in the number of previous hospitalizations [34]–[49].

Similarly to others [50], we found that cases had higher use of gastroprotective drugs than controls. As we cannot suppose that gastroprotective agents cause GI complications, rather than protect from their onset, the more likely explanation is that physicians more likely prescribe gastroprotective agents to patients with a history of GI complications, or to those at whom GI symptoms sudden occurred, i.e. to patients at higher UGIC risk.

### Strengths and limitations

Several peculiar features of our study deserve to be mentioned. First, the study is based on data from a very large unselected population, which was made possible by the fact that in Italy a cost-free uniformly organized healthcare system involves practically all citizens. By drawing out healthcare utilization data from nearly 30% of the whole Italian population, we were able to build probably one of the largest observational studies performed on the GI safety of bisphosphonates. Our data, furthermore, reflecting routine clinical practice, are unaffected by selective participation and recall bias. Second, the drug prescription databases provided highly accurate data, because reports of prescriptions by the pharmacies are essential for reimbursement and filing an incorrect report about dispensed drugs has legal consequences [51]. Third, a number of sensitivity analyses confirmed the robustness of our findings. For example, we found that neither criteria employed for UGIC diagnosis or the length of the time-window for current BPs use, affected our estimates. This further strengthens the evidence that the current use of a BP is unlikely to be causally associated with onset of serious GI complication. In addition, there was no evidence that the observed BPs - UGIC association changed by introducing lag-time periods of different lengths, thus excluding the possibility that findings were affected by “measurable” protopathic bias [26].

Our study has a number of potential limitations regarding selection bias, misclassification and confounding. Two sources of selection bias need to be considered. As outcomes were drawn from hospitalized patients, our data concerned only severe GI complications that required hospitalization. According to a large body of literature, an increased risk of mild gastrointestinal events associated with BPs use was expected [52]–[58], but we cannot detect them. It should be furthermore considered the possibility that the lack of association with stomach or duodenal complications might dilute the oesophageal risk associated with BPs.

Although patients who used BPs six months before the index date were excluded from our cohort, it is likely that prevalent users (i.e. patients who previously took these drugs) have been included. Prevalent users are patients who kept therapy over time and then are expected to be less vulnerable of experiencing the outcome of interest. Moreover, patients previously treated who already developed GI complications, when newly treated with BPs more likely took into account the recommendations for their use. These issues suggest that the inclusion of prevalent users would mask a possible positive BPs-UGIC association. We attempted to face such a problem by calculating the magnitude of the bias introduced by the inclusion of prevalent BPs users. This analysis showed that, even in the case of a strong bias (say, prevalent users currently exposed to BPs have a two-fold decreased UGIC risk than those unexposed), the true BPs – UGIC association was about that expected under the null. A higher proportion of prevalent users than that observed in the subcohort for whom such data was available, however, would have affected more strongly our estimates.

As far as misclassification is concerned, outcome as well as exposure, measurement errors would be considered. Because of privacy regulations, hospital records were not available for analysis so diagnoses cannot be scrutinized and validated. However, a high level of accuracy for most of the ICD-9 codes used to define our outcome was reported by *Cattaruzzi et al*. for the hospital discharge database similar to that used in the current study [59]. Misclassification of BPs exposure might occur because, once the drug is dispensed, it is possible that patients did not consume it. If this preferably happens when GI symptoms occur, again a protopathic bias is introduced, in this case without any possibility to control for it.

Finally, as for all observational studies, there is always some concern for residual confounding due to unmeasured factors. For example, under the assumption that use of BPs increases the UGIC risk, over-the-counter gastroprotective agents might be assumed by some patients once GI symptoms occur, so selectively reducing their outcome risk. On the other hand, the assumption of over-the-counter NSAIDs might be reduced when GI symptoms occur, again reducing the outcome risk. We attempted to face such a problem by calculating the magnitude of association with current use of BPs and risk of UGIC that a hypothetical unmeasured confounder would need to make statistically significant the BPs – UGIC association. This analysis showed that, even in the case of a highly prevalent confounder (say, over-the-counter agents would be used by the 50% of the study population) and of a strong confounder-outcome association (say, over-the-counter gastroprotective or NSAIDs would be able to cut by half and to double the UGIC risk respectively), the exposure to this factor would be strongly imbalanced between current and past use of BPs (say, more than 3.6-fold increased or reduced over-the-counter use would happen during the current use of BPs) to make significant the relationship between current use of BPs and UGIC risk. It should be noticed, however, that the confounding model employed assumes a confounder entirely independent of the factors adjusted for in the naïve analysis. If, as in our case, unmeasured confounder is strongly correlated to the confounders already adjusted for, the impact of unmeasured confounder could be much smaller than the model indicates [60].

## Conclusion

In summary, this large nested case-control study provides further evidence that current use of BPs (as alendronate and risedronate dispensed once-daily or once-weekly) for primary prevention of osteoporotic fractures is not associated with an increased risk of severe gastrointestinal complications.

## Supporting Information

Appendix S1 **Drugs and diagnoses codes used for the study purpose.** ATC and ICD9 codes used to identify the exposure of interest, the covariates and the considered diseases.
(DOC)

Appendix S2 **Independent Ethics Committees (IEC) list.** Independent Ethics Committees (IEC) list involved in the investigation.
(DOC)
