# Supplementary material for: Risk of Severe Upper Gastrointestinal Complications among Oral Bisphosphonate Users
Source: PLoS One. 2013 Dec 9;8(12):e73159. doi: 10.1371/journal.pone.0073159 (PMC3857168; doi:10.1371/journal.pone.0073159)
Supplement: Appendix S1 — Drugs and diagnoses codes used for the study purpose. ATC and ICD9 codes used to identify the exposure of interest, the covariates and the considered diseases. (DOC) [file pone.0073159.s001.doc]

### Appendix S1

| **DRUGS** | **ATC code** |
| --- | --- |
| **Exposure variables** |  |
| Alendronate | M05BA04 |
| Risedronate | M05BA07 |
| **Covariates** |  |
| Antidepressants | N06A |
| Antithrombotic | B01A |
| Gastroprotective agents (H2 receptor antagonists and Proton pump inhibitors) | A02BA and A02BC |
| Corticosteroids | H02AB |
| Statins | C10 |
| Calcium channel blockers | C08 |
| Other antihypertensive drugs | C02, C03, C07, C09 |
| Nonsteroidal antiinflammatory drugs | M01A |

| **DISEASES** | **ICD-9 CM code** |
| --- | --- |
| **Main outcome** |  |
| Ulcer, perforation and haemorrhage of oesophagus, and gastric and duodenal ulcer | 530.2x, 530.4x, 530.82, 531.x, 532.x, 533.x, 578.x |
| **First alternative outcome**: Caderette’s criteria [20] |  |
| Gastric, duodenal or peptic ulcer (acute/chronic with haemorrhage/perforation), hematemesis, blood in stool, Hemorrhage of gastrointestinal tract, unspecified | 531.0x, 531.1x, 531.2x, 531.4x, 531.5x, 531.6x, 532.0x, 532.1x, 532.2x, 532.4x, 532.5x, 532.6x, 533.0x, 533.1x, 533.2x, 533.4x, 533.5x, 533.6x, 578.0x, 578.1x, 578.9x |
| **Second alternative outcome**: EU-ADR criteria [25] |  |
| Oesophageal haemorrhage, gastric/duodenal/peptic/ gastrojejunal ulcer (acute with haemorrhage/perforation), acute gastritis with haemorrhage, atrophic gastritis with haemorrhage, other specified gastritis with haemorrhage, unspecified gastritis and gastroduodenitis, gastrointestinal haemorrhage, hematemesis, haemorrhage of gastrointestinal tract | 530.82, 531.0x, 531.1x, 531.2x, 532.0x, 532.2x, 532.2x, 533.0x, 533.00, 533.1x, 533.2x, 534.0x, 534.00, 534.1x, 534.2x, 535.01, 535.11, 535.41, 535.51, 578.x, 578.0x, 578.9x |
| **Exclusion criteria** |  |
| Osteoporotic fracture of femur, tibia, fibula and vertebrae | 733.14, 733.15, 733.16, 805.x, 808.x, 820.x, 821.x, 823.x |
| Oesophageal varices, oesophagitis, gastric, gastrointestinal, oesophageal, peptic, duodenal ulcer, gastritis and gastroduodenitis, dyspepsia, gastrointestinal haemorrhage, nausea and vomiting, Mallory-Weiss syndrome, regional enteritis | 456.0x, 530.1x, 530.2x, 530.3x, 530.4x, 530.8x, 530.9x, 531.x, 532.x, 533.x, 534.x, 535.11, 535.41, 535.5x, 535.6x, 536.2x, 536.8x, 536.9x, 537.89, 537.9x, 569.3x, 573.3x, 578.x, 787.0x, 787.1x, 787.2x, 787.3x, 789.0x, 787.x, 789.x, 530.7x, 555.x |
| Coagulation disorders | 093.0x, 286.x, 287.1x, 287.3x, 287.4x, 287.5x |
| Alcohol abuse | 305.0x |
| Chronic liver disease | 571.x |
| Cancer | 140.x-208.x |
