# Supplementary material for: Risk of Severe Upper Gastrointestinal Complications among Oral Bisphosphonate Users
Source: PLoS One. 2013 Dec 9;8(12):e73159. doi: 10.1371/journal.pone.0073159 (PMC3857168; doi:10.1371/journal.pone.0073159)
Supplement: Appendix S2 — Independent Ethics Committees (IEC) list. Independent Ethics Committees (IEC) list involved in the investigation. (DOC) [file pone.0073159.s002.doc]

**Appendix S2**

IEC Azienda Ospedaliero-Universitaria “Careggi”, Florence (Italy)

IEC Azienda Ospedaliero-Universitaria Policlinico “G. Martino”, Messina (Italy)

IEC Local Health Authority ASL Milano, Milan (Italy)

IEC Local Health Authority ASL Como, Como (Italy)

IEC Local Health Authority ASL Monza, Monza (Italy)

IEC Azienda Ospedaliero-Universitaria Policlinico “S. Orsola-Malpighi”, Bologna (Italy)

IEC Azienda Ospedaliero-Universitaria Ospedali Riuniti “Umberto I”, Ancona (Italy)

IEC Local Health Authority ASL Isontina, Friuli Venezia Giulia (Italy)

IEC Local Health Authority ASL Latina, Latina (Italy)

IEC Local Health Authority ASL Teramo, Teramo (Italy).
